# Supplementary material for: The Prevalence of Overweight, Obesity, Hypertension, and Diabetes in India: Analysis of the 2015–2016 National Family Health Survey
Source: Int J Environ Res Public Health. 2019 Oct 18;16(20):3987. doi: 10.3390/ijerph16203987 (PMC6843936; doi:10.3390/ijerph16203987)
Supplement: Supplementary file 1 [file ijerph-16-03987-s001.pdf]

Table S1. Questions used and procedures followed in identifying the presence of overweight/obesity, hypertension, and diabetes among adults under the National Family Health Survey 2015–2016.

Questions and Instructions Posted to the Respondents after Receiving Their Consent to Participate in the Study as Part of the Individual Interviews with Eligible Men Aged between 15 and 54 and Women Aged between 15 and 49	
Identification	State___________________.<br>Type of primary sampling unit: 1 = Urban, 2 = Rural.<br>Is the household selected for the state module? 1 = Yes, 2 = No (end interview).<br>If yes, what language is the questionnaire presented in?<br>01 = Assamese, 02 = Bengali, 03 = Gujarati, 04 = Hindi, 05 = Kannada, 06 = Kashmiri, 07 = Konkani, 08 = Malayalam, 09 = Manipuri, 10 = Marathi, 11 = Nepali, 12 = Oriya, 13 = Punjabi, 15 = Tamil, 16 = Telugu, 17 = Urdu, 18 = English, 19 = Garo, 20 = Khasi, 96 = Other, specify________________.
Body mass index (BMI), kg/m^2	
	Respondent's height in centimeters (cm) and weight in kilograms (kg) were measured using the SECA 213 Stadiometer (SECA Inc., Hamburg, Germany) and SECA 874 U digital scale (SECA Inc., Birmingham, UK), respectively. BMI was estimated by dividing the weight in kilograms by the height in meters squared (kg/m^2). Overweight/obesity was said to exist (and the data included in this study) if the BMI was above 25 kg/m^2. Underweight (BMI < 18 kg/m^2) and normal weight (BMI = 18–25 kg/m^2) were excluded from the analysis.
Blood pressure (BP), mm Hg	Will you allow me to measure your blood pressure? You can say yes or no to the test. You are free to decide.<br>Circle the appropriate code and sign your name.<br>1 = Granted.<br>2 = Refused<br>3 = Granted (No signature)<br>If 1 or 3 is circled, ask the following questions that may affect BP measurements:<br>Have you done any of the following things in the past 30 minutes:<br>Have you had anything to eat? Eaten: 1 = Yes, 2 = No.<br>Have you had coffee, tea, cola, or other drink that has caffeine? Had a caffeinated drink: 1 = Yes, 2 = No.<br>Have you smoked tobacco or any tobacco product? Smoked: 1 = Yes, 2 = No.<br>Have you used another type of tobacco such as ghutka, pan masala with tobacco, other chewing tobacco, or snuff? Other tobacco: 1 = Yes, 2 = No.<br>May I measure your blood pressure? I will start by measuring the circumference of your arm between the elbow and the shoulder in centimeters (cm) to make sure that I use the right equipment. Use the arm circumference measurement to select the appropriate BP monitor cuff size.<br>Circle the code for the cuff size:<br>1 = small (17–22 cm)<br>2 = medium (22–32 cm)<br>3 = large (32–42 cm)

Record the BP (systolic and diastolic) reading (three measures in all) with the help of the OMRON Blood Pressure Monitor (OMRON-healthcare, Hoofddorp, the Netherlands).

994 = Refused.

995 = Technical problems.

996 = Other.

If BP is not measured, go to the other questions.

If BP is measured 3 times with a 5-minute break between readings, circle the single number where the average diastolic and systolic measures meet:

	Average Diastolic					
**Average Systolic**	**<80**	**<85**	**85–89**	**90–99**	**100–109**	**≥110**
<120	1	2	3	4	5	6
<130	1	2	3	4	5	6
130–139	1	2	3	4	5	6
140–159	1	2	3	4	5	6
160–179	1	2	3	4	5	6
≥180	1	2	3	4	5	6

If any of the following numbers are circled:

1 = Normal (optimal)

2 = Normal (Mildly high)

3 = Normal (Moderately high)

4 = Abnormal (Mildly elevated)

5 = Abnormal (Moderately elevated)

6 = Abnormal (Severely elevated)

Note: If only two measurements were taken, record the second systolic and diastolic numbers. If only one measurement were made, record the first systolic and diastolic numbers. Abnormal BP readings (moderately to severely elevate) were included in the present study.

Blood glucose, mg/dL

Do you have any questions about the blood sugar measurement so far? If you have any questions about the procedure at any time, please ask me.

You can say yes or no to having your blood sugar measured now.

Will you allow me to take your measurement?

Circle the appropriate code and sign your name:

1 = Granted

2 = Refused

3 = Granted (No signature)

If 1 or 3 is circled, ask the following questions that may affect BP measurements:

When was the last time you had something to eat?

When was the last time you had something to drink other than plain water?

	Hours ago______<br>If less than 1 hour, record “00”<br>Record the blood glucose in mg/dL after collecting the finger-stick blood specimen and conducting a test using Optimum H Glucometer (Abbott Laboratories, Chicago, IL, USA).<br>995 = Refused<br>996 = Other<br>998 = Not tested<br>**Note:** The readings were considered equivalent to laboratory estimations of blood glucose levels made using the GlucoseOxidase method for glucose levels in the range of 10–600 mg/dL. Diabetes was said to exist (and the data included in this study) in adults who had blood glucose levels equal to or above 140 mg/dL. Those who refused and those whose blood glucose level was under 140 mg/dL were excluded.
